# Supplementary material for: SNPs in genes related to the repair of damage to DNA in clinical isolates of M. tuberculosis: A transversal and longitudinal approach
Source: PLoS One. 2024 Jun 25;19(6):e0295464. doi: 10.1371/journal.pone.0295464 (PMC11198749; doi:10.1371/journal.pone.0295464)
Supplement: S1 Table — (PDF) [file pone.0295464.s001.pdf]

Supplementary table 1. Genes related to DNA damage repair in *M. Tuberculosis*

Gene name	Identifier	Start	End	Gene length	Orientation	Essentiality
**RecF**	Rv0003	3280	4437	1158	+	nonessential
**SSBa**	Rv0054	58586	59080	495	+	nonessential
**PolD2**	Rv0269c	323338	324531	1194	-	nonessential
**MutT3**	Rv0413	499713	500366	654	+	nonessential
**XthA**	Rv0427c	516017	516892	876	-	essential
**RecD**	Rv0629c	720005	721732	1728	-	nonessential
**RecB**	Rv0630c	721729	725013	3285	-	nonessential
**RecC**	Rv0631c	725013	728306	3294	-	nonessential
**End (Nfo)**	Rv0670	769792	770550	759	+	essential
**Ku**	Rv0937c	1045199	1046020	822	-	nonessential
**LigD**	Rv0938	1046136	1048415	2280	+	nonessential
**Fpg2**	Rv0944	1053765	1054241	477	+	nonessential
**UvrD1**	Rv0949	1058260	1060575	2316	+	essential
**Mfd**	Rv1020	1138967	1142671	3705	+	nonessential
**MazG**	Rv1021	1142671	1143648	978	+	essential
**MutT2**	Rv1160	1286595	1287020	426	+	nonessential
**TagA**	Rv1210	1353522	1354136	615	+	nonessential
**UdgB**	Rv1259	1407339	1408238	900	+	nonessential
**AlkA**	Rv1317c	1477628	1479118	1491	-	nonessential
**NucS**	Rv1321	1484279	1484959	681	+	nonessential
**UvrC**	Rv1420	1594042	1595982	1941	+	essential
**DinB1**	Rv1537	1739856	1741247	1392	+	nonessential
**DnaE1**	Rv1547	1747694	1751248	3555	+	essential
**Ogt / adaB**	Rv1316c	1477134	1477631	498	-	nonessential
**PolA**	Rv1629	1830665	1833379	2715	+	essential
**UvrB**	Rv1633	1837075	1839171	2097	+	nonessential
**UvrA**	Rv1638	1843741	1846659	2919	+	nonessential
**Mpg**	Rv1688	1912979	1913590	612	+	nonessential
**RecN**	Rv1696	1919683	1921446	1764	+	essential
**Rv2119**	Rv2119	2378386	2379222	837	+	nonessential
**Cho**	Rv2191	2453819	2455756	1938	+	nonessential
**RNaseH1**	Rv2228c	2501644	2502738	1095	-	nonessential
**RecO**	Rv2362c	2643461	2644258	798	-	nonessential
**Nei1**	Rv2464c	2766859	2767665	807	-	nonessential
**SSBb**	Rv2478c	2784123	2784608	486	-	nonessential
**RuvX**	Rv2554c	2873258	2873770	513	-	essential
**RuvB**	Rv2592c	2923199	2924233	1035	-	nonessential
**RuvA**	Rv2593c	2924230	2924820	591	-	nonessential
**RuvC**	Rv2594c	2924817	2925383	567	-	nonessential
**RecGwed**	Rv2694c	3011399	3011767	369	-	nonessential
**Dut**	Rv2697c	3013683	3014147	465	-	essential
**RecX**	Rv2736c	3048562	3049086	525	-	nonessential
**RecA**	Rv2737c	3049052	3051424	2373	-	nonessential
**RNaseH2**	Rv2902c	3212162	3212956	795	-	nonessential
**MutM (Fpg)**	Rv2924c	3238601	3239470	870	-	nonessential
**RecG**	Rv2973c	3327733	3329946	2214	-	nonessential
**Ung**	Rv2976c	3332071	3332754	684	-	essential
**MutT1**	Rv2985	3342165	3343118	954	-	nonessential
**LigA**	Rv3014c	3372545	3374620	2073	-	nonessential
**DinB2**	Rv3056	3416705	3417745	1041	+	nonessential
**LigB**	Rv3062	3425584	3427107	1524	+	nonessential

**UvrD2**	Rv3198c	3569109	3571211	2103	-	essential
**AdnB**	Rv3201c	3573731	3577036	3306	-	essential
**AdnA**	Rv3202c	3577033	3580200	3168	-	nonessential
**Nei2**	Rv3297	3681320	3682087	768	+	nonessential
**DnaE2**	Rv3370c	3781501	3784740	3240	-	nonessential
**ImuB**	Rv3394c	3809442	3811025	1584	-	nonessential
**ImuA**	Rv3395c	3811022	3811636	615	-	nonessential
**RadA**	Rv3585	4026444	4027886	1443	+	nonessential
**MutY**	Rv3589	4030493	4031407	915	+	nonessential
**Nth**	Rv3674c	4115157	4115894	738	-	nonessential
**RecR**	Rv3715c	4159889	4160500	612	-	nonessential
**Prim-PolC**	Rv3730c	4180680	4181720	1041	-	nonessential
**LigC**	Rv3731	4181758	4182834	1077	+	nonessential
**MutT4**	Rv3908	4393449	4394195	747	+	nonessential

Built based on data from the Mycobrowser repository (https://mycobrowser.epfl.ch/)
